# Integrating national community-based health worker programmes into health systems: a systematic review identifying lessons learned from low-and middle-income countries

**DOI:** 10.1186/1471-2458-14-987

**Published:** 2014-09-22

**Authors:** Joseph Mumba Zulu, John Kinsman, Charles Michelo, Anna-Karin Hurtig

**Affiliations:** Department of Public Health, School of Medicine, University of Zambia, P.O. Box 50110, Lusaka, Zambia; Umeå International School of Public Health (UISPH), Umeå University, Umeå, SE 90185 Sweden

**Keywords:** National community-based health worker programmes, Integration, Health systems, Low- and middle-income countries

## Abstract

**Background:**

Despite the development of national community-based health worker (CBHW) programmes in several low- and middle-income countries, their integration into health systems has not been optimal. Studies have been conducted to investigate the factors influencing the integration processes, but systematic reviews to provide a more comprehensive understanding are lacking.

**Methods:**

We conducted a systematic review of published research to understand factors that may influence the integration of national CBHW programmes into health systems in low- and middle-income countries. To be included in the study, CBHW programmes should have been developed by the government and have standardised training, supervision and incentive structures. A conceptual framework on the integration of health innovations into health systems guided the review. We identified 3410 records, of which 36 were finally selected, and on which an analysis was conducted concerning the themes and pathways associated with different factors that may influence the integration process.

**Results:**

Four programmes from Brazil, Ethiopia, India and Pakistan met the inclusion criteria. Different aspects of each of these programmes were integrated in different ways into their respective health systems. Factors that facilitated the integration process included the magnitude of countries’ human resources for health problems and the associated discourses about how to address these problems; the perceived relative advantage of national CBHWs with regard to delivering health services over training and retaining highly skilled health workers; and the participation of some politicians and community members in programme processes, with the result that they viewed the programmes as legitimate, credible and relevant. Finally, integration of programmes within the existing health systems enhanced programme compatibility with the health systems’ governance, financing and training functions. Factors that inhibited the integration process included a rapid scale-up process; resistance from other health workers; discrimination of CBHWs based on social, gender and economic status; ineffective incentive structures; inadequate infrastructure and supplies; and hierarchical and parallel communication structures.

**Conclusions:**

CBHW programmes should design their scale-up strategy differently based on current contextual factors. Further, adoption of a stepwise approach to the scale-up and integration process may positively shape the integration process of CBHW programmes into health systems.

## Background

Many low- and middle-income countries (LMICs) are facing human resources for health (HRH) shortages [1–3]. According to the World Health Organization (WHO), more than 57 countries face critical health worker shortages, of which the majority (63%) are in sub-Saharan Africa [1]. This shortage has affected the delivery of health services, and has also hindered progress towards attainment of the health-related Millennium Development Goals [4]. Causes of health workforce shortages include the inability of countries to train, retain and distribute health workers [4, 5]. The involvement of community–based health workers (CBHWs) in primary health care is one strategy of addressing this gap [6].

The term CBHW is broad in scope and includes home-based care providers, community health workers, community-based treatment supporters, and traditional birth attendants [6].

Although some countries had already started engaging CBHWs in delivering primary health care before 1978, the number of countries increased further following the Declaration of Alma Ata in 1978 [2, 6, 7]. Article VII.7 of the Declaration recognised CBHWs as being vital to improving access to primary health care. The document stated that primary health care “relies, at local and referral levels, on health workers, including physicians, nurses, midwives, auxiliaries and community workers as applicable, as well as traditional practitioners as needed, suitably trained socially and technically to work as a health team and to respond to the expressed health needs of the community” [8], p 2.

However, there was a decline in interest in CBHW programmes in the late 1980s [9]. The reduced interest in the programmes resulted from the challenges that the first CBHW programmes experienced, and which reduced their programmatic effectiveness [2, 3, 6]. Specific difficulties included inadequate training, remuneration or incentives; limited supervision; deficient continuing education opportunities; inadequate supplies and medicines; and limited recognition or acceptance by other health workers [2, 3, 7]. Severe economic crisis faced by a number of countries also contributed to the reduced interest in CBHW programmes [9].

Nonetheless, in the early 1990s, renewed enthusiasm for CBHW programmes in LMICs emerged [2, 6]. Several issues precipitated this interest, one of them being the increased advocacy by the WHO on the role of task shifting as a means of reducing the burden on overstretched health care systems [10]. Task shifting involves reviewing and delegating tasks away from clinical staff to non-clinical staff such as CBHWs, thereby enabling clinical staff to concentrate on their specific areas of expertise [1, 11, 12]. In addition, the demands imposed by the growing HIV epidemic, other infectious diseases, non-communicable diseases, and general health coverage inequalities especially in rural communities, contributed to this renewed interest in community-based health care [6, 13]. Further, an increase in the number of countries adopting decentralised health care policies and strategies, as well as community partnership policies, also contributed towards this renewed interest [2].

In an attempt to increase the potential for delivering positive health outcomes at a large scale, there was a move towards implementing national CBHW programmes [6, 10]. Compared to small scale CBHW programmes (e.g. those implemented locally by Non-Governmental Organisations), Liu et al. [6] suggest that large-scale programmes have the potential to deliver positive health outcomes if appropriate attention is given to ensuring that they have strong management systems. In addition large scale CBHW programmes have the potential to successfully and rapidly recruit, train, and deploy a large cadre of CBHWs. Further, the specification of duties, standardisation of incentives, and the supervision as well as training which characterise most of these programmes should also facilitate CBHWs’ ability to deliver good health services [2, 3, 14].

In order to be effective and sustainable at national scale, Singh [15], p20 suggests that CBHWs should be “integrated into a nationwide primary health care system through recognition in national health care planning, regulation and implementation. Parallel systems for community health that are not integrated with the primary health care system risk weaker referral systems, supervision and support by facility based care providers, and policymaker buy-in to support supply chain and other systems components”. However, many national CBHW programmes have faced considerable difficulties in the process of shifting from small-scale local projects to national CBHW schemes, with the lack of integration into the national health system being one of the major problems encountered [10]. Studies on some national CBHWs programmes have shown that their integration into their respective health systems has not been optimal [6, 16, 17].

Although there has been an increase in the number of countries developing national CBHW programmes, there is limited systematic documentation on the factors that influence the integration process of CBHWs into health systems. Recent systematic reviews on CBHWs have focused more on their role in improving disease-specific outcomes [18–21], as well as factors affecting the implementation of CBHW programmes for maternal and child health [2]. This paper intends to fill this knowledge gap by systematically assessing the factors that may influence the integration of these programmes into health systems in LMICs. We focus on the integration of existing CBHW programmes into official training, supervision and civil service systems, as well as the acceptability of the CBHWs to other health workers and the community. We expect that it will provide useful information that may guide integration processes in countries which are currently implementing similar programmes, as well as in those which intend to develop such programmes.

### Conceptual framework

**Figure 1:**
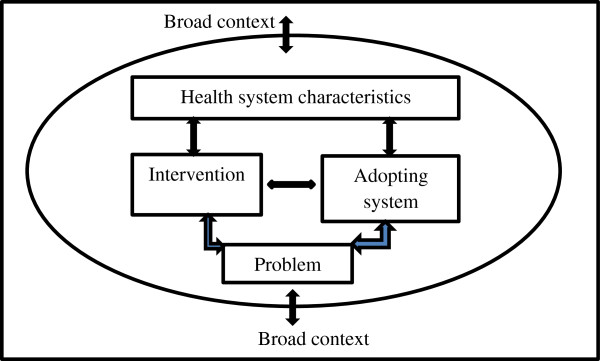
**Conceptual framework for analysing integration process (adopted from Atun et al.** [22] **).**

In analysing the factors that influence the integration process of national CBHWs into health systems, we have adopted a conceptual framework from Atun et al. [22]. According to this framework, integration of new health interventions into health system functions is influenced by the ***nature of the problem*** being addressed, the ***intervention****,* the ***adoption system***, the ***health system characteristics,*** and the ***broad context*** (Figure 1). Drawing from this conceptual framework, we developed the following assumptions: First, the **nature of the problem**, such as the magnitude and discourse about the impact of and solutions to the HRH gap at national and global level, may influence actors’ perspectives towards CBHW programmes, and these in turn may shape their integration process. Second, the **attributes of the intervention**, such as quality of service delivery by national CBHWs, may also influence the integration process. Integration may also be influenced by the level of the programme’s compatibility with **health system characteristics**, such as resources and regulatory systems, as well as the **broader context** which includes, for example, demographic, economic, political and socio-cultural factors. Finally, the perspectives of national CBHW programmes by actors within the **adopting system** – who include policy makers, organisations, health workers, patients and communities – may either facilitate or inhabit the integration of CBHWs in health systems.

We selected this conceptual framework because it enables analysis of the interactions and interconnections between various factors influencing the integration process, thereby “allowing a systematic and holistic analysis of adoption and diffusion of health interventions in general” [22], p106.

We define integration as the process or extent and pattern of acceptability and adoption of the health intervention – in this case a CBHW programme – into critical functions of a health system [23–26]. These include the existing governance and leadership of an existing national health system as well as the shared goals and outcomes of existing health activities.

Meanwhile, the integration status of health interventions into health systems can take different forms, which include being fully, partially or not integrated with different elements of the health system [22–24]. Table 1 below shows how we have defined the terms “fully integrated”, “partially integrated”, and “not integrated” in this paper.

**Table 1:** **Definition of integration status**

	Integration status		
Selected health systems elements [24]	Full integration	Partial integration	Not integrated
Governance and leadership	Management and supervision of CBHWs is conducted by other health workers and institutions in the ministry of health	Management and supervision of CBHWs is not completely conducted by other health workers and institutions in the ministry of health. Private stakeholders such as NGOs are also involved	CBHWs do not receive any supervision from other health workers and institutions in the ministry of health
Financial resources	CBHWs are part of the civil service and are paid standardised monthly salaries by the government	CBHWs are not part of civil service, but receive standardised incentives from the government	CBHWs are not part of the civil and do not have standardised incentives from the government
Human resources	CBHWs receive standardised training from the ministry of health and are fully accepted as well as supported by other health workers	CBHWs receive standardised training from the ministry of health but are not fully accepted by some health workers	CBHWs do not receive any form of standardised training from the ministry of health and are not recognised by other health workers
Service delivery	CBHWs perform standardised tasks; stakeholders recognise, accept and utilise the services provided the CBHWs	CBHWs perform standardised tasks; but some stakeholders do not recognise, accept and utilise the services provided the CBHWs	CBHWs do not have standardised tasks and duties
Population	CBHWs are recruited from the community and are recognised and accepted by the community	CBHWs are recruited from the community but are discriminated or not accepted by part of the community	Not all CBHWs are recruited and work within their community and most community members do not recognise or accept CBHWs
Outcomes and Goals	CBHW services and duties are in line with the national primary health care system	CBHW services and duties are not in line with all of issues contained in the national primary health care system	CBHW duties and services are not developed based on the national primary health care system

## Methods

### Study design

We carried out a systematic review to examine factors that may influence integration of national CBHW programmes, using Atun’s [22] framework as a lens for analysis. A systematic review is a literature review focused on a research question or objective that tries to identify, appraise, select and synthesize quality research evidence relevant to the question or objective. In conducting the review, we were guided by the description by van der Knaap et al. [27] and Petrosino et al. [28] of the main aspects of a systematic review. These include formulation of a research question or objective; determination of the inclusion and exclusion criteria; description of the search for potential studies; screening of relevant studies that have been identified for eligibility according to the inclusion and exclusion criteria; determination of the quality of the selected studies; and production of data extracts, analysis and interpretation of the results.

### Search strategy

We systematically searched the following websites for literature about national CBHW programmes between November 2013 and March, 2014: CINAHL, Medline, PubMed, ScienceDirect, Web of Science, BioMed Central, and the Cochrane Collaboration. For a programme to qualify to be included in the study, it had to meet the following criteria: the programme must have been formed and operated by the government; it should have training, supervision and incentive structures that are standardised and well-defined by the government; it should have been scaled nationally in or after the 1990s (the period when there was renewed enthusiasm for CBHW programmes in LMICs); and it should have been in operational for not less than five years. Only four programmes met the inclusion criteria: Accredited Social Health Activists (ASHAs) in India, Community Health Agents (CHAs) in Brazil, Health Extension Workers (HEWs) in Ethiopia, and Lady Health Workers (LHWs) in Pakistan. Other large CBHWs that did not fit within this inclusion (for example the Bangladeshi programme, with about 80,000 CBHWs, and that was initiated and is operated by BRAC, a national Bangladeshi NGO) were excluded from the study.

Having selected the studies, we then searched the websites using specific programme names as follows: “The Community Health Agents in Brazil”, or “Health Extension Workers in Ethiopia”, or “Accredited Social Health Activists in India”, or “The Lady Health Workers in Pakistan”. Relevant literature was also identified by checking references of the articles and the websites of the WHO. A total of 3410 documents were identified, as reflected below in Table 2.

**Table 2:** **Search outcomes for literature about national Community-Based Health Worker programmes**

Data Source	Countries				
	Brazil	Ethiopia	India	Pakistan	Total
The Cochrane Collaboration					48
Web of science	5	43	21	31	100
PubMed	245	38	13	58	354
Medline	8	40	35	44	127
Biomed Central	194	56	45	39	334
CINAHL	32	9	2	18	61
Science direct	15	1115	33	350	1513
References and WHO websites	426	243	37	167	873
Sub Totals	925	1544	186	707	

### Study selection and quality assessment

To ensure inclusion of relevant, high quality papers in this review, our inclusion criteria for documents comprised: peer-reviewed publications only; conducted in Brazil, Ethiopia, India and Pakistan; and including a focus on the integration of national CBWH programmes into health systems. We included papers with different study designs, including qualitative, mixed-methods, reviews, and programme evaluations.

**Figure 2:**
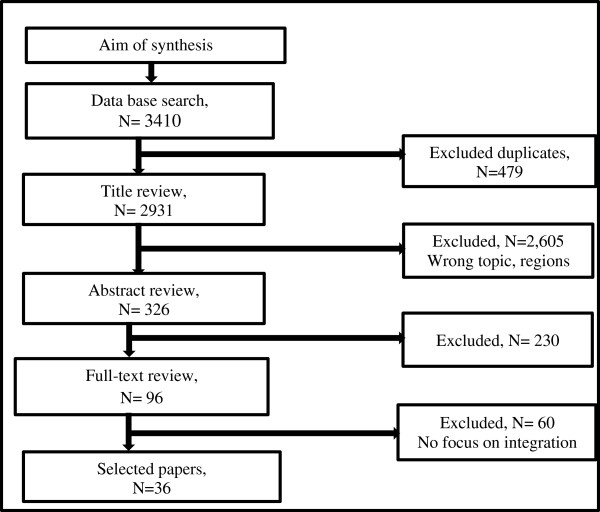
**Search strategy and paper selection flow chart.**

With these inclusion criteria in mind, we then followed the Preferred Reporting Items for Systematic reviews and Meta-Analyses (PRISMA) guidelines by Moher et al. [29] in selecting the studies. In accordance with the guidelines, we first excluded all duplicates (479) from the 3,410 search outcomes initially identified. Then we reviewed all the titles of the remaining 2,931 research papers and reports, of which we excluded 2,605, because they focused either on the wrong topic or region or both. We then remained with 326 outcomes. Subsequently we retrieved and assessed the abstracts of the 326 papers, of which we excluded 230 because they did not address the subject of integration of CBHWs into health systems. Finally, we retrieved 96 full-length papers that were shortlisted after abstract review, in order to screen them in accordance with the inclusion criteria. At this stage, we also subjected the papers to the main elements of the Critical Appraisal Skills Programme (CASP) quality assessment that has been used to appraise studies, and especially those that use qualitative approaches [30]. This process resulted into the final 36 papers as shown in Figure 2.

The Critical Appraisal Skills Programme (CASP) quality assessment tool has been used in other syntheses of primarily qualitative evidence, such as Munro [31] and Glenton et al. [2]. Below is an overview of the quality criteria we used:

- Are the research questions or objectives clearly stated?
- Is the approach appropriate for the research question?
- Is the study context clearly described?
- Is the role of the researcher clearly described?
- Is the sampling method clearly described?
- Is the sampling strategy appropriate for the research question?
- Is the method of data collection clearly described?
- Is the data collection method appropriate to the research question?
- Is the method of analysis clearly described?
- Is the analysis appropriate for the research question?
- Are the claims made supported by sufficient evidence?

### Data analysis

We analysed and synthesised data in the 36 selected papers using a thematic analysis approach. This is “a method for identifying, analysing and reporting patterns (themes) within data. It organizes and describes data set in detail and goes further to interpret various aspects of the research topic” [32], p 79. Thematic analysis is one of the data analysis approaches recommended by the Cochrane Qualitative Review Methods Group [2, 33]. The first step involved familiarisation with the included studies. During this process, themes regarding factors that could enable and/or inhibit the integration process were inductively developed, based on key components of the conceptual framework on integration of innovations into health systems [22]. The themes were then separately reviewed by all authors, after which final agreement on the themes (as given in Table 3) was achieved.

**Table 3:** **Selected factors influencing the integration process**

Integration condition	Factors influencing integration process
Characteristics of the problem	Human Resource for Health problem
	Discourse about Human Resource for Health problem
	Discourse about CBHWs
Attributes of the intervention	Service delivery
	Performance of CBHWs
Adopting system	Politicians and professional health workers programme perceptions
	Community members programme perceptions
Health system characteristics	Training systems for CBHWs
	Supervision process for CBHW
	Incentive structure for CBHWs
Broad context	Demographic factors
	Economic factors
	Socio-cultural factors

Having developed the themes, we described pathways on how and why factors relating to the nature of the problem, intervention, adoption system, health system characteristics, and the broad context may influence the integration of national CBHW programmes into health systems. This was necessary because the national CBHW programmes are introduced into health systems with dynamic and complex feedback loops, alongside non-linear relationships which extend beyond the health system and which are intricately linked to the context within which the system is embedded [22].

## Results

In this section, we present our findings on the factors that influence the integration of national community-based health worker (CBHW) programmes into health systems. The section has been organised around the major components of the conceptual framework on integration of health interventions into health systems, namely: characteristics of the problem, attributes of the intervention, the adoption system, and the health system characteristics. The fifth component of the framework, broad context, has been discussed within the other four components of the framework (the characteristics of the problem, attributes of the intervention, the adoption system, and the health system characteristics). The section starts by outlining the characteristics of the studies included in the review, followed by a description of the integration status of the four national CBHW programmes included in the paper into their respective health systems.

### Study Characteristics

Thirty six (36) studies were included for the final review. Of these studies, thirteen (13) were reviews, ten (10) were mixed-methods studies, eight (7) were qualitative, while the remaining six (6) were programme evaluations. More detailed characteristics of the different studies, their aims and major findings, are provided in Table 4.

**Table 4:** **Study characteristics**

N o	1 ^st^Author/Year [Citations]	Country	Study type/ design	Type of CBHWs	Aim	Key issues/findings
**Brazil specific studies**						
1	Svitone [46]	Brazil	Review	CHAs	To document primary health care lessons from the Northeast of Brazil following the implementation of CHAs Programme	Comprehensive information available, a decline in infant mortality, a rise in immunization, and timely interventions in times of crisis
2	Macinko [35]	Brazil	Program Evaluation	CHAs	To assess the effects of an integrated community-based primary care programme on microregional variations in infant mortality (IMR), neonatal mortality, and post-neonatal mortality rates from 1999 to 2004	Results show that infant mortality rate declined about 13 percent from 1999 to 2004, while Family Health Program coverage increased from an average of about 14 to nearly 60 percent
3	Aquino [16]	Brazil	Program Evaluation	CHAs	To evaluate the effects of the Family Health Programme (FHP) on infant mortality at a municipality level	The FHP had an important effect on reducing the infant mortality rate in Brazilian municipalities from 1996 to 2004. The FHP may also contribute toward reducing health inequalities
**4**	Zanchetta [40]	Brazil	Mixed methods	CHAs	To assessing the effectiveness of CHAs' actions in situations of social vulnerability	Barriers to CHAs' effectiveness included professional powerlessness, communication gaps, fragmented teamwork, organizational and structural barriers
**Ethiopia Specific studies**						
**5**	Girma [40]	Ethiopia	Review	HEWs	To understand implications of strategies for human resource development (HRD) by 2015	The process to develop policy and strategy for managing human resource for health has been started
**6**	Negusse [26]	Ethiopia	Mixed methods	HEWs	To document the initial community perspectives on the Health Service Extension Programme in Welkait	HEWs are helpful, HEWs are more preferred over TBAs, HEWs provide good health services. Limitations: less visits, poor knowledge on major communicable diseases
**7**	Teklehaimanot [41]	Ethiopia	Qualitative	HEWs	To assess the working conditions of HEWs in Ethiopia and their job satisfaction	Health indicators have improved, performance in skilled delivery and postnatal care not satisfactory. Limited quality of service, utilization rate, access, referrals and programme evaluation
**8**	Admassie [47]	Ethiopia	Program Evaluation	HEWs	To evaluate the short-term and intermediate-term impacts of the HEW programme on child and maternal health indicators in the programme villages	The proportion of children and women using insecticide-treated bednets for malaria protection are significantly larger in programme villages than in non-programme villages
**9**	Koblinsky [52]	Ethiopia	Review	HEWs	To explore Ethiopia’s progress toward achieving MDG 5 through the Health Extension Programme	Achieving the set targets is a daunting task despite reaching the physical targets of two health extension workers per health post
**10**	Damtew [53]	Ethiopia	Qualitative (Case study)	HEWs	To examine conditions that may affect the quality of HEWs training in Ethiopia	Training inadequacies
**11**	Medhanyie [58]	Ethiopia	Mixed methods	HEWs	To investigate the Knowledge and performance of the HEWs on antenatal and delivery care as well as the barriers and facilitators to service provision	Poor knowledge of HEWs, poorly equipped health posts, and poor referral systems affected acceptability of services
**12**	Medhanyie [59]	Ethiopia	Program Evaluation	HEWs	To assess the role of HEWs in improving utilization of maternal health services in rural areas in Ethiopia	Better utilization of family planning, antenatal care etc. Limited contribution to health facility delivery, postnatal check-up etc.
**13**	Birhanu [61]	Ethiopia	Mixed methods	HEWs	To assess mothers’ experiences and satisfaction with health extension service	Most mothers had good relationships, were satisfied with and had positive attitude towards HEWs. Programme was however criticized for not including curative services and the less attention given to static services at health post
**14**	Teklehaimanot [42]	Ethiopia	Qualitative (Case study)	HEWs	To describe the strategies, human resource developments, service delivery modalities, progress in service coverage, and the challenges in implementing the HEP	Health system reformed to create a platform for integration/ institutionalization of the HEP with appropriate human capacity, infrastructure, and management structures
**India specific studies**						
**15**	Scott [49]	India	Qualitative (Case study)	ASHAs	To investigate the contextual features hindering the ASHAs' capacity to increase quantitative health outcomes and act as cultural mediators and agents of social change	SHAs limited by: (1) the outcome-based remuneration structure; (2) poor institutional support; (3) the rigid hierarchical structure of the health system; and (4) a dearth of participation at the community level
**16**	Gopalan [60]	India	Mixed-methods	ASHAs	To examine the performance motivation of community health workers (CHWs) and its determinants on India's Accredited Social Health Activist (ASHA) programme	Performance motivation mainly influenced by the individual and the community level factors, while the health system factors scored the least
**17**	Kumar [43]	India	Program Evaluation	ASHAs	To study the factors influencing the work performance of ASHAs in community	Limitations included less knowledge, caste system, limited incentive practices and inadequate incentives
**18**	Shrivastava [54]	India	Mixed-methods	ASHAs	To evaluate the knowledge, attitudes and practices of ASHA workers in relation to child health	Gaps still exists in ASHAs’ knowledge regarding various aspects of child health morbidity
***Pakistan specific studies***						
**19**	Afsar [50]	Pakistan	Program Evaluation	LHWs	To estimate the proportion of patient referral and to identify the factors associated with unsuccessful referral in Karachi, Pakistan	Limited communication and counselling skills of LHWs contributed to significant proportion of unsuccessful referrals
**20**	Afsar [51]	Pakistan	Qualitative	LHWs	To assess the strengths and weakness of the National Programme for Family Planning and Primary Health Care from the LHWs’ perspectives	Strengths: Some community members accepting LHWs. Weaknesses: contractual job, low salaries, irregular payment, no career development and poor logistical support
**21**	Douthwaite [38]	Pakistan	Mixed methods	LHWs	To evaluate the Lady Health Worker programme	The LHWP has succeeded in increasing modern contraceptive use among rural women
**22**	Haq [57]	Pakistan	Mixed methods	LHWs	To evaluate job stress among community health workers in Pakistan	Challenges: stress, low socio-economic status, long distances; inadequate, medical supplies, stipends, communication skills, lack of career structure
**23**	Haq [39]	Pakistan	Qualitative	LHWs	To document the perceptions of LHWs on their knowledge and communication needs, image building	Many respondents described their communication skills as moderately sufficient. Knowledge on emerging health issues was insufficient
**24**	Hafeez [37]	Pakistan	Mixed methods	LHWs	To review the LHW programme and explore various aspects of the process to extract tangible implications for other similar situations	Improved community links with first level care facilities, earned community trust. Limitations: poor support from sub-optimal health facilities, financial constraints and political interference
**25**	Mumtaz [55]	Pakistan	Mixed-methods	LHWs	To explore the impact of socio-cultural factors on LHWs' home-visit rates	Performance is constrained by both gender and biradari/caste-based hierarchies.
**26**	Wazir [17]	Pakistan	Review	LHWs	To conduct a SWOT analysis of the National Program for Family Planning and Primary Health Care in Pakistan	*Strengths*: comprehensive planning, implementation and supervision mechanisms, selection and recruitment processes. *Weaknesses:* slow progress, poor program integration, job insecurity and delayed salaries
**Studies focusing on more than one country**						
**27**	Hermann [44]	Ethiopia and others	Review	HEWs	To investigate whether present CBHW programmes for ART are taking into account the lessons learnt from past experiences and analyse the extent to which they are seizing the new ART-specific opportunities	Adequate remuneration key to CBHW retention. Sufficient attention to be given to supervision, continuous training and health systems strengthening
**28**	Celletti [34]	Brazil, Ethiopia, etc.	Qualitative	CHAs HEWs	To evaluate the contribution of CHWs with a focus on identifying the critical elements of an enabling environment that can ensure that they provide quality services in a manner that is sustainable	Important requirements include adequate systems integration, political commitment; good planning, definition of scope of practice, selection, educational issues, career path, registration, licensure and certification; recruitment and deployment; adequate remuneration, supervision; referral system; supplies
**29**	Kane [19]	Brazil, Ethiopia, India, Pakistan, *etc.*	Realist synthesis (Review)	CHAs, HEWs, ASHAs, LHWs	To explore if randomised controlled trails could yield insight into the working of the interventions, when examined from a different perspective, a realist perspective	Positive mechanisms: anticipation of being valued; perceived improved social status; sense of relatedness with the health system; increased self esteem, sense of self efficacy, enactive mastery of tasks; sense of credibility, legitimacy
**30**	Lewin [20]	Brazil, Ethiopia, India, Pakistan, *etc.*	Systematic review	CHAs, HEWs, ASHAs, LHWs	To assess the effects of LHW interventions in primary and community health care on maternal and child health and the management of infectious diseases	LHWs provide promising benefits in promoting immunisation uptake and breastfeeding, improving TB treatment outcomes, and reducing child morbidity and mortality when compared to usual care
**31**	Liu [6]	Brazil, Ethiopia, India, Pakistan, *etc.*	Review	CHAs, HEWs, ASHAs, LHWs	To explore CBHW programmes that have been deployed at national scale, as well as scalable innovations found in successful nongovernmental organization-run community health worker programmes	Ability by national CBWH programmes to reach scale is impressive, but quality and management challenging. If well managed programmes integrated into a well-functioning primary healthcare system can promote care and act as an effective link
**32**	Wouters [48]	Ethiopia and others	Synthetic review	HEWs	To review the impact of community-based support services on ART delivery and outcomes in resource-limited countries	CBHWs are not necessarily cheap or easy, a good investment to improve coverage of communities in need of health services
**33**	Jaskiewicz [56]	Ethiopia Pakistan and others	Review	HEWs LHWs	To review the influence of work environment in increasing community health worker productivity and effectiveness	Essential elements for improving productivity: defined workload, supportive supervision, supplies and equipment, and respect from the community and the health system
**34**	Balabanova [45]	Ethiopia, etc.	Review	HEWs, etc.	To discuss why some countries or regions achieve better health and social outcomes than others at a similar level of income and to show the role of political will and socially progressive policies	Attributes of success included good governance, political commitment, effective bureaucracies, ability to innovate and adapt to resource limitations, the capacity to respond to population needs and build resilience into health systems to face challenges. Transport infrastructure, female empowerment, and education also played a part
**35**	Glenton [2]	Brazil, Ethiopia, India, Pakistan, *etc.*	Systematic review	CHAs, HEWs, ASHAs, LHWs	To explore factors affecting the implementation of LHW programmes for maternal and child health	Barriers and facilitators were mainly tied to programme acceptability, appropriateness and credibility; and health system constraints
**36**	Perry [3]	Brazil and others	Review	CHAs	To summarize the history, recent evolution, and current evidence of the effectiveness of CHWs around the world	CBHWs promote healthy behaviors, extend reach of health systems, help address health workforce resources shortage, and reduce health disparities

### Integration status of national community-based health worker programmes

Below we introduce the four selected national CBHW programmes, and present a summary of the extent and pattern of their integration into their health systems. Overall there was considerable variation across and within the programmes with regard to their integration (Table 5).

**Table 5:** **Integration status of national CBHW programmes**

	Name of CBHW programme and integration status			
Health systems elements [24]	CHA-Brazil [3, 16, 31, 35, 36]	LHWs-Pakistan [3, 17, 37–39]	HEWs –Ethiopia [3, 26, 34, 40–42]	ASHAs- India [3, 6, 43]
Governance and leadership	Full integration	Full integration	Full integration	Partial integration
Financial resources	Full integration	Full integration	Full integration	Partial integration
Human resources	Partial integration	Partial integration	Full integration	Partial integration
Service delivery	Partial integration	Partial integration	Partial integration	Partial integration
Population	Full integration	Partial integration	Partial integration	Partial integration
Outcomes	Full integration	Full integration	Full integration	Full integration
Goals	Full integration	Full integration	Full integration	Full integration

With regard to operational status, the CHAs operate within the Brazil’s Family Health Strategy [16], LHWs in the National Programme for Family Planning and Primary Health Care [17], HEWs in the Health Extension Programme [3, 26, 40] and ASHAs in the Rural Health Mission programme [3]. More details about the national CBHW programmes (their roles, type of incentives and mode of supervision) are provided in Table 6.

**Table 6:** **Summary of national scale programmes**

Country	CBHW programme	Roles	Incentives	Supervision
Brazil	Community Health Assistants (CHAs)	Promoting breastfeeding as well as providing prenatal, child care, immunizations, screening and treatment of infectious diseases services	From $100 to $228 per month	- Done through family health care teams
	- About 240,000 CHAs			- Teams consist of nurses and physicians from the local clinics
	- Launched in 1991			- 33,000 family health care teams
Pakistan	Lady Health Worker (LHWs)	Supporting maternal and child health services, which include family planning, HIV/AIDS and treatment of minor illnesses. Providing health education, essential drugs for minor ailments, contraceptives, vaccination and making referrals	$343 per year	Conducted by Lady Health Worker supervisor
	- About 90,000 LHWs			
	- Launched in 1992.			
Ethiopia	Health Extension Workers (HEWs)	Providing basic first aid, contraceptives, and immunizations, as well as diagnosing and treating malaria, diarrhoea, and intestinal parasites	About $84 monthly	Conducted by district team comprising health officer, a public health nurse, an environmental technician and health education expert
	- About 34,000 HEWs			
	- Launched in 2003			
India	Accredited Social Health Activists (ASHAs)	Community mobilisation, motivating women to give birth at health posts, promoting immunisations, family planning, treating basic illness, keeping demographic records, and improving village sanitation.	About 600 rupees ($10) for facilitating an institutional delivery, and 150 rupees ($2.50) for each child that successfully completes immunisation session	Conducted by ASHA facilitators
	- About 800,000 (ASHAs)			
	- Launched in 2005			

### Factors influencing integration of national community-based health workers into health systems

#### Characteristics of the problem

In the first of the major factors relating to the integration process that have been identified in the conceptual framework, we focus on how the characteristics of the problem (in this case the human resources for health crisis) may influence the acceptability and adoption of the intervention that has been designed to solve the problem (i.e. the national CBHW programme) within health systems.

#### Human resources for health problem and discourse

The HRH gap prevailing in most LMICs, including Brazil, Ethiopia, India and Pakistan, has precipitated increased attention and interest by policy makers and politicians in implementing and integrating national CBHW programmes into their national health systems [2, 3, 6, 20, 44, 45]. The early discourse relating to the HRH gap in Brazil, Ethiopia, India and Pakistan focused mainly on expanding health facilities and training highly skilled health workers [2, 3]. However, these approaches proved difficult due to limited capacity to train and retain highly skilled health workers in the countries [3]. This limited capacity generated increased interest by international and national institutions in continuing discussions about the effects of, as well as potential solutions to the health workforce crisis. As the discourse evolved, there was recognition that there may not be enough professional health workers available within an acceptable time frame [3]. On this basis, the discourse about addressing the HRH gap shifted towards developing national CBHW programmes.

Further, increased discussion and advocacy by actors within the global context, such as the WHO, also motivated countries to implement CBHW programmes [2]. For example “being signatory to Alma Ata declaration, the Government of Pakistan took concrete steps in collaboration with WHO, and launched its first nation-wide CBHW programme known as Lady Health Worker’s Programme in 1994” [17], p 3. In general, the increased demand for primary health care, as well as the disease burden in Brazil, Ethiopia, Pakistan and India also facilitated the acceptance of national CBHWs into the health systems [6, 35, 38, 41, 46–48].

More recently, “the growing focus on the human resource crisis in health care has re-energised debates regarding the roles that CBHWs may play in extending services to ‘hard to reach’ groups and areas, and in substituting for health professionals for a range of tasks” [2], p 4. In this context, national CBHWs are thought to play an important role in achieving demonstrable health benefits that are directly related to the health-related Millennium Development Goals, namely reducing child malnutrition, reducing child and maternal mortality, and controlling HIV/AIDS, tuberculosis (TB) and malaria [2, 3]. Such positive discussion and views about the possible roles of CBHWs in efforts towards achieving the Millennium Development Goals has positively influenced the integration process of national CBHWs into governance and health service delivery [2, 6, 19, 20, 48].

In addition, the WHO has continued, as part of efforts to address the global health worker shortage, to recommend implementation of CBHW programmes [49]. Specifically, in 2010, the Global Health Workforce Alliance (GHWA) organised the Global Consultation on Community Health Workers, and recommended for the integration of CBHWs into national health systems. Part of this integration process was to include a regular and sustainable remuneration stipend for CBHWs. The GHWA, which is under WHO, is an innovative partnership aimed at coordinating solutions to the global health workforce crisis, and has a membership of over 400 organisations.

However, this pathway towards integration - the HRH crisis and its associated discourse - has been limited in a number of ways. In order to reach national scale, some countries have rapidly scaled up or deployed national CBHW programmes in a relatively short period of time. The Pakistan and Indian programmes, for example, deployed 90,000 and 462,000 CBHWs respectively over the last decade, while the Ethiopian HEW programme deployed 34,000 workers over a period of four years. This rapid scale-up of CBHW programmes generated several challenges in terms of quality and management of the programme [16, 40, 41, 49–55]. These challenges were often due to insufficient and inconsistent programme funding, and inadequate programme logistics management. Further, the programmes were sometimes poorly planned which resulted in problems of sustainability in terms of both quality of care and retention of health workers. These problems resulted in a lack of continuity in the relationship between CBHWs and their communities, thereby affecting the acceptability and adoption of the CBHWs in population component of the health systems [6].

### Attributes of the intervention

This section focuses on the pathway between attributes of the intervention (the national CBHW programme) and the integration process of the CBWHs in the health system.

#### National community-based health workers’ ability to deliver services

Perceived relative advantage of national CBHWs’ programmes in terms of service delivery over other similar programmes, such the traditional birth attendants, can positively influence the integration process. High quality service delivery may be triggered in situations where national CBHWs see their incentives as consistent, predictable, appropriate and fair in relation to their tasks, as well as where they have a reasonable workload, good training, and regular supervision from professional health workers [2, 6, 19, 34–36, 44, 46, 47, 56]. These components can increase CBHWs’ “willingness and ability to deliver services, which in turn can lead to better quality services and to improved health outcomes” [2], p 38. Good services and improved health outcomes may generate increased interest among actors in the adopting systems towards CBHWs, which can subsequently enhance acceptability and adoption of national CBHWs by the population. For instance, in Ethiopia “ninety three per cent of participants indicated that they would prefer HEWs to assist them during labour, rather than traditional birth attendants”, as they perceived HEWs as being more knowledgeable [26], p 3.

This pathway – of good services or performance facilitating the integration process – may, however, be threatened in many ways. Limited availability and accessibility to supplies and medicines by CBHWs can affect service delivery [16, 41, 49, 52, 57, 58] and subsequently the acceptability and adoption of CBHWs within the population and health service delivery functions of the health systems. In Pakistan, limited access to drugs by LHWs for community activities “caused embarrassment and made Lady Health Workers suspect in the eyes of the community, because they were accused of selling drugs and contraceptives in the market” [51], p 5. In Ethiopia, inadequate facilities at some health posts for giving deliveries discouraged some women from using the services promoted and provided by HEWs [41, 42, 58, 59]. In India, challenges at health posts made promoting of institutional births by the ASHAs less acceptable, such that their advice to women to go to the clinic proved unsound, and an ASHA risked *“*losing face in the community and people were less likely to trust her on other matters” [49], p 1610. In the community, their inability to always provide drugs “induced the community’s non-confidence on ASHAs” [60], p 9.

Failure by training programmes to adequately cover all relevant skills can affect CBHWs’ ability to deliver services, and this can subsequently undermine their acceptability and adoption of the programme both by other health professionals and the population. In India, because of limited training for curative services, the ASHAs were more “identified as ‘link workers’ or facilitators for appropriate care and the community have less acceptance for their curative role. The ASHAs were less confident on their curative care skills” [60], p 9. In addition, effective service delivery by ASHAs was constrained by the incentive structure. The ASHAs could not perform some tasks as they were done at a net personal financial loss. Scott & Shanker [49], p 1609 explain that the ASHAs complained that ”by the time they are fully immunized, we have spent almost Rs. 500 [in transportation costs] on the child and we get only Rs. 150 as compensation”. The ineffective, outcome-based payment structure also constrained ASHAs’ work output [43], since some ASHAs tended to focus more on those activities which attract payment, such as provision of contraceptives and facility-based deliveries, at the expense of other essential activities such as general health promotion activities at community level [6, 43, 49]. However, there is also evidence that gender inequality and poverty made some of the ASHAs to see the post of a health worker as a path to 'liberation', and they willingly put in extra effort when conducting their duties, despite not receiving adequate remuneration [43].

### Adopting system

In the third pathway, we analyse how and why the perspectives and participation of the actors in the adopting systems (e.g. politicians, decision makers, policy makers, other health workers, and community members) can influence the acceptability and adoption of national CBHW programmes into health systems.

A positive perspective by some politicians and community members in the adoption system towards national CBHW programmes, which may be triggered by their involvement in the programme, can facilitate integration. Full participation of actors in the adoption system can facilitate integration process as it can lead actors to view the CBHWs as “legitimate and credible, to have confidence in their knowledge and skills and to view their services as relevant and valuable. This in turn can lead to good relationships between CBHWs and recipients” [2], p 38.

#### Perspectives by politicians and professional health workers

In Ethiopia and Pakistan positive perspectives of national CBHW programme by politicians facilitated integrated governance and leadership resulting in common goals and standardised financial resources. The Prime Ministers in both countries spearheaded the launch of the programmes, thereby facilitating the integration process of CBHWs into national civil service structures [17, 42, 45]. For instance in Pakistan, Wazir et al. [17], p 2 explain that “it is heartening to see that the LHW programme received adequate political commitment, since 1994. There has been a wide recognition of the programme among the political arena and all government quarters. The financial and administrative support has continued without any interruption”.

On the other hand, negative perspectives of national CBHWs by other health workers can also affect the integration process. Negative perspectives can be triggered by a lack of proper definition of CBHWs’ tasks, such as in the cases of Brazil and Pakistan. In Brazil, “CHAs faced resistance to acceptance as from other health professionals (mainly nurses)—due to issues of liability, unclear roles, their ambiguous position in the entrenched physician/nurse-based hierarchy and overlap with work assigned to auxiliary nurses” [16], p 332. Physicians’ perspectives and minimal opportunities to communicate directly with CHAs were the major challenges given by CHAs’ regarding their integration within Family Health System teams [16]. In Pakistan, non-acceptance by the established professions was also cited as one of the issues which affected the integration process of Lady Health Workers into the health system [17].

#### Community perspectives

Positive perspectives of national CBHWs by community members, which may be triggered through recruitment of local people, can facilitate the integration process into the population. In Pakistan, “the hiring of local people contributed towards the high level of acceptability and trust that LHWs enjoyed in communities, as well as an increased community acceptance of several culturally sensitive issues like family planning” [51], p 3. Selection of local people in both India and Brazil also positively influenced the acceptability and adoption of national CBHW services. In Brazil, since they live in the community, CHAs were able to provide “24 hours of basic health services to the community at the doorstep”, a process which is greatly appreciated, “especially by women, who, for cultural reasons could not leave their houses” [16], p 339.

Apart from just the concept of recruiting local people, the sex of CBHWs also plays a role in influencing the integration process. Studies in Ethiopia attributed part of an increase in mothers’ utilisation, acceptability and adoption of the services provided by HEWs to the recruitment of local females [50, 61]. Female workers “were preferred for the premises of degree of closeness, easier disclosure of personal problems and as a matter of cultural norms. Associated with cultural norms and biological factors, mothers are ready to share their personal issues to females than to males” [61], p 6. In India, recruitment of local women as ASHAs helped in increasing use of modern contraceptives among women in the communities [38].

This pathway between positive perspectives by actors leading to enhanced integration may be undermined in many ways. First, integration may be affected if the community members that are selected as CBHWs or the structures or leaders that offer support to the CBHWs are regarded as poorly functioning or otherwise suspect [2]. This can affect integration as community members may not perceive the CBHW programme as credible or trustworthy. For example, in some instances, inadequate consultation between local leaders and community members in India resulted into the selection of ASHAs with limited community support [49, 60].

Further, cultural perspectives of gender, which include not supporting in particular young and unmarried women to walk and do the work, can also affect the integration process [50, 17]. In Pakistan, “if one of the LHWs was married and the other unmarried, the married one provided respectable company and was better able to discuss family planning issues, a topic that was generally not acceptable for the unmarried worker to raise” [55], p 55. In addition, in some parts of Pakistan, “the LHWs found it difficult to talk to men about family planning” [2], p 27. The LHW’s mobility, especially discussing with unrelated males on sensitive issues, led to a loss of status of women, as such a practice was considered as a sign of being uncultured [39, 55]. Overall, acceptance of services can be limited due to gender discrimination. “Even though the program’s focus is maternal and child health care, LHWs still have to face difficulties owing to traditional gender norms in many parts of the country” [17], p 5.

Caste-based village hierarchies may also affect the integration process by restricting mobility of CBHWs. In Pakistan, the caste system discouraged visits by LHWs beyond the biradari (extended family) boundaries. Since most LHWs tended to be from lower castes, they preferentially visited co-members of their extended family who are likely to share similar socioeconomic circumstances, thereby resulting in other social classes not fully accessing the health services [17, 55, 57]. A study by Mumtaz et al. [55], p 54 confirmed that “the rejection of their services by higher caste women also discouraged LHWs from venturing to their homes”. In India, the lower caste ASHAs were discriminated against by the higher class people, thereby affecting service delivery, as well as the acceptability and adoption of services within the affected areas [43].

Further, the receipt by CBHWs of monthly salaries may trigger mixed responses at community level, which in turn may affect the integration process. While the CBHWs may feel that “their incentives are appropriate, community members may question the motivation and credibility of CBHWs who are salaried rather than volunteers” [2], p 18. In India, the ASHAs were perceived as being entirely accountable to auxiliary nurse midwifes, especially since they collect their remuneration from the primary health centre [49].

### Health system characteristics

Finally, we analyse the pathway between the health systems characteristics and integration of national CBHWs into health systems. Review of data suggests that integration of national CBHWs into existing structures may enhance programme compatibility with local practises, values and regulations. Compatibility with health systems characteristics can foster good relationships between CBHWs and other professional health workers, while also improving referral processes, information transfer, and general health service delivery. A combination of these components may enhance the pattern and extent of acceptability and adoption of national CBHWs by actors into the adopting system.

Examples of how integration in health systems characteristics can facilitate programme compatibility can be drawn from Brazil, Ethiopia and Pakistan. In Brazil, integration of the CHA programme within the municipal health council (responsible for allocating financial resources for health, which come from the federal and state governments) enhanced acceptability and adoption of the CHAs by the government officials, health service providers and other citizens. This process also guaranteed allocation of finance for primary health care to the CHA programme as well as acceptability and adoption of CHAs within the family health teams [16, 34]. The integration of CBHW training components into Ministries of Health in Brazil and Ethiopia facilitated compatibility of training and certification of national CBHWs with those of institutions responsible for developing training curriculum, conducting training and certifying professional health workers [3, 16, 34, 37]. In Pakistan, supervision is done by senior LHWs who are accountable to professional health workers [17, 37]. Further, placing CBHWs in the civil structure in Brazil, Ethiopia and Pakistan facilitated provision of standardised monthly incentives which helped national CBHWs to deliver services, thereby positively contributing towards the integration process [16, 17, 34, 37, 51]. In India, integration of the ASHA programme into the ASHA mentoring group and National Health Services Resource Center helped to reduce resistance towards ASHAs from health workers as well as from community members. These institutions played a crucial role in taking various steps aimed at resolving the differences at the national level [43, 49, 60].

The pathway that we propose, linking health systems characteristics and the integration of national CBHWs into health systems, may be challenged in a number of ways. For example, the existence of parallel or hierarchical communication structures can limit the integration process of national CBHWs into the functions of the health system, by affecting the smooth flow of information. In India, the hierarchical communication structure in place constrained information flow as well as any ability by the programme to address key health systems challenges [49]. The ASHAs, who at times tended to have far more understanding than many health professionals of the dynamics affecting the use of health services (for example, why many women choose not to give birth in the hospital despite their being offered incentives for institutional deliveries), could not effectively relay the message to the decision makers due rigid communication systems [49, 60]. In Pakistan, studies have shown that “there is a management information system but it is not integrated with the overall health system, which leaves a vacuum in decision making because the problems and issues from the grass root level are not taken into consideration while making allocations, disbursements, procurements etc. [Although] there are good linkages at higher levels, at the field levels (i.e. basic health units and other areas), the linkages are poor due to inherent weaknesses in the health system itself” [17], p 4.

## Discussion

Given that this systematic review was structured around five components – namely the nature of the problem, the intervention, the adoption system, the health system characteristics, and the broad context - the discussion is thus centred around these themes.

Several factors dictated the degree and nature of national CBHW programme integration into different countries’ health systems. In Brazil, integration of the CHA programme into the municipal health councils facilitated compatibility of the programme with the governance, financial, and service delivery health systems functions. Similarly, in India, integration of the ASHAs programme into the National Health Services Resource Center helped in reducing conflicts between ASHAs and other actors in the health system. In Ethiopia and Pakistan, positive perspectives by politicians facilitated full integration of CBHWs into the civil service structure and Ministry of Health in general. Integration of the programme into the Ministries of Health in Brazil, Ethiopia and Pakistan, facilitated full integration of the training processes of the CBHWs by national training institutions. Further, in all the four countries, positive perspectives by some community members, which were triggered mainly though participatory processes, positively facilitated the integration process.

On the other hand, limited definition of tasks at health facility level in Brazil affected acceptability of CHAs by other health workers, thereby resulting into sub-optimal or partial integration into the human resources function of health system. Similarly, discrimination in Pakistan and India, based on social-cultural practices, gender, age and marital status affected the full integration process of CBHWs into the population as well as the health service delivery functions of the health system. In Ethiopia, limited service delivery affected integration of some of the services provided by HEWs within the population function of the health system. In India the hierarchical communication structure resulted into rigidity and top-down power which constrained information flow and subsequently affected the integration process of ASHAs.

**Figure 3:**
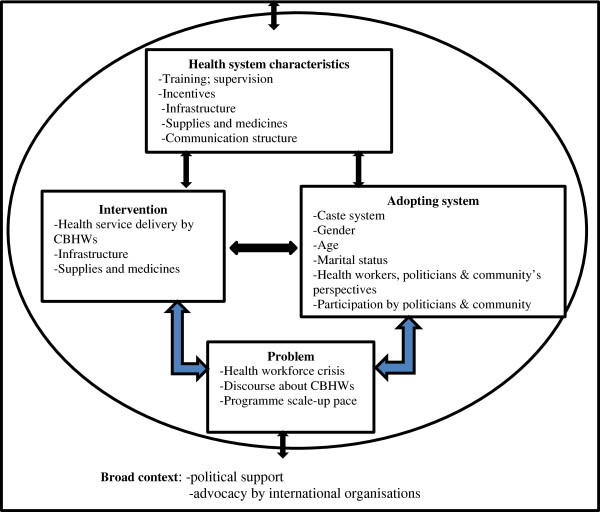
**Factors influencing integration of national CBHWs in health systems.**

In general, we found no instance when the national CBHW programmes are fully integrated into health systems. Instead, different aspects of the programmes are integrated in different ways, whether fully or partially into one or more functions of the health systems (Table 5). This ‘mosaic’ type of integration is indicative of a complex, multi-functional and dynamic process. For example, while some aspects of the CHA programme in Brazil are fully integrated into the governance and financial functions of the health system, other aspects are partially integrated into the human resources and health service delivery health systems functions. Similarly, while the incentive aspect of the LHW programme in Pakistan is fully integrated into the financial functions, other aspects of the programme are only partially integrated into the population, health service delivery, and human resources health systems functions, with the management information system being completely non-integrated. While the programme is fully integrated into some components of the health system in Ethiopia, it is only partially integrated in health service delivery. In India, the ASHAs are fully integrated in the goals and outcomes of the health system functions but only partially integrated in the other functions.Based on the findings, we built on the original conceptual framework of integration of national CBHWs into health systems to include the specific factors which affect the integration process as reflected in Figure 3.

Studies on other health innovations have indicated that for integration to be successful, the adopting system or context should be motivated and competent, in terms of skills, resources, values, goals, regulations [22, 62–64]. Limited capacity within a given context to adopt CBHWs can push the CBHWs to the margins of the health system, where they may occupy an ambiguous position as volunteers/workers. Lack of clarity in the CBHW position may result in CBHWs being expected to work regular hours and yet not having the employment rights of other health workers. This situation may affect health service delivery as well as programme sustainability, as CBHWs may feel unfairly treated [10].

On the other hand Schneider et al. [10], p 186 argue that “it is also not clear that the solution to the difficulties associated with CBHWs lies in incorporating them wholesale into the civil service”. These authors suggest that placing all CBHWs within the civil services may limit the possibilities for more for inclusive participation. Second, this can also limit the expression of a range of different motivations or non-financial incentives such as clearly defining responsibilities, appropriate job aides, resources/supplies, and community connectedness [14, 47, 65–68]. Promoting community connectedness is vital as it is a cornerstone of the CBHW model [6, 35]. Community connectedness is also relevant for the integration process as it may promote “a sense of relatedness with the local public health services, and thus accountability towards the system, a sense of credibility and legitimacy of being part of the local public health services, an anticipation of being valued by the local public health services as well as an assurance that there is a system for back-up support” [19], p 5. Putting more emphasis on placing CBHWs in the civil service structure as compared to promoting community connectedness may affect acceptability and adoption of national CBHWs at community level, since it may trigger mixed perspectives regarding motivation, credibility and accountability of CBHWs. Some people may perceive CBHWs as being too accountable to the Ministry of Health as opposed to the community, as was the case in India [2, 49].

Having successful national CBHW programmes is likely to require strategies that ensure the integration both at the community and formal health system levels. Further, to reduce the challenges related to planning and management as a result of rapid scale-up, it may be necessary for countries to adopt a stepwise approach during the integration process. Such an approach can provide sufficient space for drawing lessons to guide programme scale-up.

### Strengths and limitations

One main strength of the study lies in the extensive search of the literature. The inclusion of papers utilising different methodological approaches, including mixed-methods papers and reviews, provided in-depth insights into factors influencing the integration of national CBHWs into health systems. The use of a multi-disciplinary team (with expertise in both public health and anthropology) in the review, and the synthesis of data, enriched the synthesis as it provided us with an opportunity to draw and collate team members’ interpretations of the findings. However, one of the limitations of the review was the possibility of missing out some publications. We tried to mitigate this by conducting several searches between November 2013 and March 2014, and also searching the references of publications. The other limitation was the inclusion of only studies conducted in English as this is the main language with which the authors were conversant. However, the inclusion of systematic reviews helped us capture some of studies that we may have missed out due to language barriers, thereby helping us to develop a rich account of the factors influencing the integration of national CBHWs in LMICs.

## Conclusion

Our findings indicate that different aspects of the national CBHW programmes are integrated in different ways within particular functions of the health systems. The acceptability and adoption of national CBHW programmes in health systems was shaped by the interaction between the perspectives of the actors within the adopting system, as well as the compatibility of CBHWs with the health systems characteristics and the broad context. Further the perspectives of the human resources for health gap by actors in the adopting system and within the broad context affected the integration process. Prior understanding of contextual factors that can impact integration process is critical for successful integration of national CBHW programmes into health systems. This suggests the need for having comprehensive approaches for providing baseline contextual data at the beginning of an integration process, about which stakeholders should be aware as they integrate CBHW programmes into the health systems. In addition, it is important to follow a stepwise approach to any national CBHW programme integration process in order to reduce some of the planning and managerial difficulties that can be associated with rapid scale-up and integration.

As they mature, CBHW programmes have the potential for contributing towards reducing the huge human resources for health gap and extending primary health care services to ‘hard to reach’ groups and areas if appropriate attention is given to their integration processes in the health systems. Considering that health systems are interconnected, dynamic and complex in nature, and that they consist of independent agents whose behaviour is based on physical, psychological and social rules, the use of other research frameworks which acknowledge health systems as complex and adaptive systems would help provide additional insights on this subject.

## Abbreviations

ASHA: Accredited social health activist
CBHW: Community based health worker
CHA: Community health agent
HEW: Health extension worker
LHW: Lady Health worker.
